# Mobile Apps Aimed at Preventing and Handling Unintentional Injuries in Children Aged <7 Years: Systematic Review

**DOI:** 10.2196/45258

**Published:** 2023-09-06

**Authors:** Annett Schulze, Ann-Kathrin Lindemann, Fabian Brand, Johanna Geppert, Axel Menning, Paula Stehr, Doreen Reifegerste, Constanze Rossmann

**Affiliations:** 1 Department of Risk Communication German Federal Institute for Risk Assessment Berlin Germany; 2 Department of Media and Communication Ludwig-Maximilians-University Munich Munich Germany; 3 Department of Prevention and Health Promotion Bielefeld University Bielefeld Germany

**Keywords:** mobile health, mHealth, caregiver, parental, prevention, first aid, pediatric, review method, injuries, health app, needs, mobile phone

## Abstract

**Background:**

Despite various global health crises, the prevention and handling of unintentional childhood injuries remains an important public health objective. Although several systematic reviews have examined the effectiveness of different child injury prevention measures, these reviews did not address the evaluation of mobile communication intervention tools. Whether and how mobile apps were evaluated provides information on the extent to which communication theories, models, and evidence-based knowledge were considered. Previous studies have shown that the effectiveness of mobile apps increases when theories and evidence are considered during their development.

**Objective:**

This systematic review aimed to identify research on mobile apps dealing with the prevention and handling of unintentional injuries in children and examine the theoretical and methodological approaches thereof. In addition, this review analyzed the different needs of various target groups of the mobile apps described in the articles.

**Methods:**

In total, 8 electronic databases, ranging from interdisciplinary to medical and technical as well as social sciences databases, were searched for original research articles or brief reports in peer-reviewed journals or conference proceedings. Moreover, this review encompassed a systematic scan of articles published in the *BMJ* journal *Injury Prevention*. These steps were followed by a snowball search based on the literature references in the articles identified through the initial screening. The articles had to be written in English or German, published between 2008 and 2021, and evaluate mobile apps dealing with the prevention and handling of unintentional child injuries. The identified 5 studies were analyzed by 5 independent researchers using an inductive approach. Furthermore, the quality of the studies was assessed using the *Mixed Methods Appraisal Tool*.

**Results:**

A total of 5 articles were included and assessed with regard to overall quality of theoretical and methodological foundations, assessed variables, the focal app’s architecture, and the needs of the study participants. The overall study quality was moderate, although part of this classification was due to a lack of details reported in the studies. Each study examined 1 mobile app aimed at parents and other caregivers. Each study assessed at least 1 usability- or user experience-related variable, whereas the needs of the included study participants were detailed in only 20% (1/5) of the cases. However, none of the studies referred to theories such as the Technology Acceptance Model during the development of the apps.

**Conclusions:**

The future development and evaluation of apps dealing with the prevention and handling of child injuries should combine insights into existing models on user experience and usability with established theories on mobile information behavior. This theory-based approach will increase the validity of such evaluation studies.

## Introduction

### Background

The risk of unintentional injuries in children has repeatedly been revealed as a relevant factor in medical treatment. The second wave of the German Health Interview and Examination Survey for Children and Adolescents (KiGGS Wave 2, 2014-2017) showed that, under the consideration of previous studies [1], unintentional injuries in children remain at a continuously high level, especially in times of changing conditions and associated shifts in risk. For example, at the start of the COVID-19 pandemic, physicians initially predicted a higher risk of unintentional poisoning incidents because of the increased use of cleaning agents and disinfectants [2], which was later reported in studies from several countries (Italy [3], Brazil [4], United States [5], Iran [6], Australia [7], and Morocco [8]). Shifting risks and associated consequences for the well-being of children clearly underline the importance of research and measures to prevent unintentional child injuries.

Unintentional injuries vary by age and encompass various categories, such as traffic injuries (eg, motor vehicle–, bicycle-, and pedestrian-related accidents), burns, fires, falls, drowning, suffocation, and poisonings [9,10]. Most of these injuries are preventable [11]. However, to know how such injuries could be prevented, this information must reach the respective target groups via appropriate communication measures, for example, via mobile apps. Apps may play a decisive role by providing tools, information, and practical guidance to prevent, mitigate, and handle the risk of unintentional injuries [12]. Thus, research on apps proves to be crucial to develop appropriate, informative, and user-friendly communication measures.

In the context of preventing unintentional injuries in children aged <7 years, Stehr et al [13] provided a detailed analysis of intervening communication strategies and their effectiveness. The authors emphasized that the chosen communication was more effective when a theory was applied in the study. Highlighting the potential of digital media, the research also showed that tailoring improves the effectiveness of digital health communication interventions. The researchers concluded that digital adaptation is an important aspect of disseminating knowledge and informing those who can take measures to prevent unintentional child injuries: “caregivers, i.e., parents and other guardians as well as childcare workers and health professionals working with children” [13].

Given these facts, the successful development of an app depends on several factors, which are described in the following section.

### Theory- and Evidence-Based Digital Communication Through Mobile Apps

Mobile health (mHealth) technology such as mobile apps can be an effective communication tool to distribute health information [14,15]. Apps offer a number of advantages: they not only are an instrument of mass communication but also enable the provision of (tailored) information on, for example, preventive measures in 1 communication channel [16]. The information can be received anywhere, at any time, and in any situation. To be effective, the information must be evidence-based and evaluated [17]. The term *evidence-based* refers conventionally to evidence-based medicine being defined as “the conscientious, explicit and judicious use of current best evidence in making decisions about the care of individual patients” [18]. As Larson [17] noted, most of the existing mHealth apps lack evidence when it comes to the intervention itself. However, this evidence-based health information is necessary to be able to make evidence-informed decisions [19].

When making decisions based on information provided in an app, several aspects need to be considered: (1) the content of the information; (2) the presentation of the information (eg, design and layout); and (3) from the user’s point of view, how comprehensive and relevant the information is [19] or what the user’s needs are when searching for information (eg, length of text, features, and different modes of communication such as visualization). Thus, the term evidence-based refers not only to proven scientific findings in the respective medical context. It also includes scientific insights into which communication channel, which format, and which message work effectively for which target group [20], as well as data that allow for conclusions to be drawn on the structure and presentation of the app’s content.

In addition, communication strategies that apply a communication theory such as the Theory of Planned Behavior are more effective in producing certain outcomes (eg, knowledge improvement). However, and in relation to prevention, Stehr et al [13] showed that almost two-thirds of their analyzed communication strategies were not theory based. Health communication theories allow for the prediction and explanation of individual behaviors, such as the use of a particular means of communication and the acceptance related to it. Therefore, not only the content itself but also the way it is presented as well as the health information behavior of the target groups play a decisive role in the development and use of apps.

To date, little is known about the usability of mobile apps regarding child injury prevention [21,22] or their effectiveness (for mHealth in general, see the study by Chib and Lin [23], and for child injury prevention, see the studies by Cho et al [24] and Ning et al [25]). After a systematic review of the literature on unintentional injuries in people of different ages, Omaki et al [22] concluded that evaluation data on smartphone apps are lacking. Furthermore, they could not provide any insights into the role that theory plays in the development of these digital intervention tools [22]. Similar to the findings of Stehr et al [13], only a few of the included studies described the theoretical background. Omaki et al [22] suggested that a more detailed description of the theories is necessary to be able to make generalizable statements on the role that theory plays in changing safety behaviors through the use of an app.

### Technology Adoption

Changing safety behaviors, disseminating knowledge, and increasing self-efficacy are all important outcome types when communication on prevention is researched. However, the precondition for the effectiveness of an app is its use. Thus, it is not only about adapting protective behavior but also about adapting media use behavior to expedite technology adoption. However, this depends on aspects such as the perceived ease of use or perceived usefulness of the app, its perceived trustworthiness, and perceived risks regarding data protection [26]. In addition, previous research shows that trust in the app publisher and content [25] as well as in the technology itself [27] increases the acceptance of an app.

There are few studies that have developed and evaluated mobile apps for child health promotion (eg, based on the Theory of Planned Behavior [28]) and systematic reviews on educational aspects of mobile injury prevention programs [29]. However, there is to date no systematic review on the theory-related evaluation of mHealth interventions focusing on child injury prevention and handling.

### Objectives

This study identified research on mobile apps dealing with the prevention and handling of unintentional injuries in children and sought to gain insights into the different theoretical and methodological approaches of the identified studies. Furthermore, our systematic review tried to understand the different needs of various target groups of these mobile apps and, thus, took a user-centered design approach into account. These objectives are specified in our research questions (RQs): (1) Which apps for the prevention and handling (first aid) of unintentional injuries in children have been studied in scientific literature? Which app architecture characteristics and features can be described? What do they visualize? Who is their target audience? (RQ 1); (2) What theories and methods are used to evaluate these mobile apps? (RQ 2); and (3) Which needs do private caregivers, professional caregivers, and health professionals have when using apps dealing with the prevention and acute (pain) management of unintentional injuries in children aged <7 years? What constraints may prevent the use of the apps? (RQ 3).

## Methods

### Preparatory Steps

Before we carried out our systematic search, we conducted a cursory review and prereview mapping of articles dealing with mobile apps aiming at the prevention or acute (pain) management of unintentional injuries. This step seemed necessary not only to identify possible keywords for the definition of an appropriate search string but also to gain insights into the field of mobile app research. This prereview also helped specify our RQs and identify relevant databases and journals. Thus, our systematic literature review followed the steps comprehensively described by Xiao and Watson [30] and was conducted in accordance with the PRISMA (Preferred Reporting Items for Systematic Reviews and Meta-Analyses) guidelines [31] and checklist (Multimedia Appendix 1). A research protocol was registered in the PROSPERO database (ID omitted for double-blind peer review).

### Search Strategy

Regarding academic databases, we included the technical-oriented databases *IEEE Xplore* and *ACM Digital Library*; the multidisciplinary databases *Scopus* and *Web of Science*; and databases from the disciplinary fields of psychology (*PsycINFO*), communication sciences (*Communication Abstracts* and *Communication & Mass Media Complete*), and biomedical and life sciences (*PubMed*) in our search. Using a Boolean approach, the search string combined the terms “app” or “application” with different synonyms for “children” and several unintentional injury-related categories such as “drowning,” “poisoning,” or “accidents” derived from the definitions offered by Sleet [9] and Schnitzer [10] (RQ 1) and the results of our prereview mapping of relevant articles. The search string also encompassed several usability-related categories such as “ease of use” or “user experience” derived from our research interest (RQs 2 and 3). Research has shown that the needs and preferences of users are highly correlated with perceived usability [32], whereas usability-oriented app research might also indicate factors or constraints that limit or prevent the use of an app. Finally, as our search string (Multimedia Appendix 2) aimed at yielding relevant articles on the prevention and acute (pain) management of injuries, we decided to exclude research on “disease self-management” apps.

Furthermore, we decided to conduct a systematic scan of articles published in the *BMJ* journal *Injury Prevention*. The added value of complementary hand searching has repeatedly been marked in scientific discussion [33,34] as it reduces the possibility of missing relevant studies. The separate search for relevant articles published in *Injury Prevention* seemed to promise a useful addition to our search strategy, not only because of the journal’s specific focus on the prevention of unintentional injuries as well as its broad scope with regard to injuries in general but also because our prereview mapping yielded particularly interesting results from the aforementioned journal [22].

### Inclusion Criteria

Several inclusion and exclusion criteria were defined to evaluate the identified papers for further consideration in the literature review (*eligibility assessment*). For inclusion, studies had to have been written in English or German, had to elaborate on the elements of the main RQs, and had to have been published between the years 2008 and 2021. We chose this specific time frame as digital apps became increasingly prevalent with the release of the first *iPhone* in 2007 and, more specifically, with the launch of the Apple App Store in 2008 [35]*.* Following the logic of the research process, the search in the databases could only include articles up to and including March 2021—this was the first point of data collection, whereas the hand search covered all articles up to and including the end of April 2021.

The population of interest was health professionals and private and professional caregivers of children aged <7 years. We chose this age because, first, unintentional injuries in the home and during recreational activities are most common at this age [36]. Second, infants, toddlers, preschoolers, and school beginners depend on the knowledge of caregivers as they need to learn which risks to take and which to avoid [37,38]. If there was no exact age specification for the children and no explicit specification of an age of >6 years, the studies were included. For an overview of all the inclusion criteria, see Multimedia Appendix 3.

### Exclusion Criteria

Articles that did not meet the inclusion criteria were excluded. Furthermore, articles describing mobile apps that dealt with the handling and prevention of intentional child injuries (eg, abuse) or tracking and collecting individual health data as part of an ongoing treatment were excluded, as were studies that did not provide empirical data, merely focusing on technical or theoretical aspects. For an overview of all the exclusion criteria, see Multimedia Appendix 3.

### Systematic Database Search

A total of 358 papers were retrieved from 8 academic databases. Duplicate removal reduced our sample to 229 papers that went through several assessment and selection stages. The selection process is presented in the PRISMA flowchart (Multimedia Appendix 4) as well as in the overview in Multimedia Appendix 2.

Given the number of identified papers, 5 raters were involved in the eligibility assessment. To ensure a coherent and consistent selection process, we validated our interrater reliability. We randomly selected 100 papers from our sample. The titles and abstracts of these papers were assessed by all 5 raters. The Fleiss κ*,* an adaptation of the Cohen κ for ≥3 raters [39,40], was used to analyze our interrater agreement. The Fleiss κ accounts for chance agreement between several coders and measures not only accuracy (eg, by aligning the coding with the codebook) but also precision (eg, ensuring that agreement between different coders is not due to chance alone [41]). Measurement is carried out based on a calculated score, whereby a score of 1 indicates perfect agreement and a score of 0 indicates agreement totally owing to chance. After assessing the randomly selected papers, we calculated a Fleiss κ value of *0.63—*implying substantial agreement [42]. In view of the number of raters, we judged the level of agreement to be acceptable to assess the titles and abstracts of the remaining articles by only 1 rater each.

The remaining 129 articles were equally distributed among the 5 researchers involved in the title and abstract check. Each article was assessed by 1 researcher. This procedure left us with 11 papers. To confirm eligibility, these papers were further assessed through a full-text screening by 2 raters each. This step particularly focused on the documented objectives as well as the target users of the mobile apps described in the respective articles. Finally, discrepancies or disagreements concerning a particular eligibility assessment were resolved through discussion and consensus-oriented decision-making by all researchers.

The systematic database search ultimately yielded a total of 4 articles that were considered relevant for our literature review.

### Systematic Scan of Injury Prevention (BMJ Journal) Articles

A total of 899 papers were retrieved from volumes 14 to 27 of this journal. The articles were reviewed for eligibility by 2 researchers. Owing to the quantity of articles, they were assessed first based on the title and then on their abstracts. Again, critical cases were discussed within the team, and a final decision was reached by consensus. Initial title analysis led to the exclusion of 95.9% (862/899) of the papers. The following review of the remaining articles’ abstracts further excluded 97% (36/37) of the papers. Full-text screening of the remaining paper finally resulted in the inclusion of 1 article.

### Reference Tracking

The final step of our literature review included a (backward) snowball search based on the literature references of all 12 papers included in the full-text screening. Following Wohlin [43], snowballing is “particularly useful for extending a systematic literature study,” offering options to find additional studies that were not detected in the searched databases. Thus, 330 articles were further reviewed for eligibility by 2 researchers. Title and abstract analysis led to the exclusion of 97.3% (321/330) of the articles, so that 9 articles remained for the subsequent full-text screening. However, none of these articles met the inclusion criteria. As previously, critical cases in this step were discussed within the team.

As a result of these 3 different search strategies, 5 articles were selected for our comprehensive literature review.

### Assessing Quality

To critically appraise the quality of the selected studies, we used the *Mixed Methods Appraisal Tool* (*MMAT*), which was developed for the appraisal stage of complex systematic literature reviews [44]. The MMAT specifically allows for the quality assessment of different study designs in systematic mixed study reviews, enabling us to assess the quality of all 5 included studies at once. Conflicting assessments were discussed between 2 researchers involved in this systematic literature review and finally resolved through consensus. The final appraisal and evaluation of the included studies are explained in more detail in the *Results* section.

### Extracting, Analyzing, and Synthesizing Data

All 5 articles were coded using the qualitative data analysis tool MAXQDA (VERBI Software GmbH). The code system (Multimedia Appendix 5) was developed using an inductive approach; based on the original articles, we identified dimensions, categories, and subcategories that described the apps as well as their evaluation process and the major findings. The entire article was coded, including graphics and information boxes. A total of 5 coders worked on the material—coded, discussed, and recoded—striving for coherence through an iterative process. The coding process involved 4 stages. First, 2 coders coded the same article on their own. Second, they discussed the marked overlaps and divergences. If an intercoder agreement was not achieved, the whole team reviewed and reflected on the relevant passage (“negotiated agreement” [45]). Third, the codes were refined if necessary. Fourth, coding was repeated. Owing to the small number of studies and their various differences regarding the applied methods and data analysis strategies, we did not carry out any statistical evaluations. In addition, the data were analyzed using Miro (RealtimeBoard Inc dba Miro), a web-based whiteboard that allowed us to group different codings collaboratively.

## Results

### Characteristics of the Included Studies

Table 1 provides an overview of the app and study characteristics of the included studies.

**Table 1:** Overview of the included studies.

Study	App characteristics					Study characteristics				
	Name	Objectives	Main focus	Target audience	Research questions or objectives		Consideration of previous research	Participants (sample size)	Data collection	Data analysis
Iskander et al [46]	Dental Trauma first aid mobile app	Deliver information on dental trauma	Handling	Parents	Comparison of the effectiveness and user preference of the app with a poster containing the same information and testing of the app’s suitability to inform parents about dental trauma management		Cursory overview of literature on peer-reviewed apps	Caregivers (n=89): any adult accompanying a child to the participating dentist offices	Survey (conducted via a laptop or tablet)	Statistical techniques, for example, cross-tabulations and independent chi-square statistics
Jones et al [47]	GUS^a^	Support parents and carers in reducing unintentional injuries	Prevention	Parents and caregivers	Parents’ assessment of the usability of the app (ease of use, usefulness, and esthetics), consideration of multiple perspectives on the app, and identification of strengths and weaknesses of the app as well as potential for improvement		Scoping literature review on unintentional childhood injuries and review of app stores, websites, and apps providing information on unintentional injuries	Mothers (n=15)	3 focus groups	Thematic analysis
Litovitz et al [48]	webPOISONCONTROL app	Increase access to accurate information on poisoning, reduce costs of poison centers and increase their efficiency, and harmonize triage	Handling	The public, parents, and employees of poison centers	Assessment of the feasibility and user acceptance of the app and safety and correctness of triage recommendations		Not mentioned	App users (n=1339 web-based feedback respondents and n=9256 consecutive, public webPOISONCONTROL cases) and a toxicologist (n=1)	Web-based survey, app use data, and reported cases	Statistical techniques and quality assurance review—manual verification of each case
Richmond et al [49]	Active & Safe Central	Provide web-based sport injury and prevention information	Prevention	The public, coaches, parents, athletes, injury researchers, and practitioners	Description of the development of the web-based tool, collection of feedback on the selection of information for the app, and user experience of the app (data visualization and interactivity)		Systematic literature review on sport and recreational activities and their injury risks and review of websites and apps providing information on sports injuries	End users (n=16)	2 focus groups, including app use data	Not mentioned
Roberts et al [50]	Make Safe Happen	Help parents and caregivers make their homes safer by identifying injury risks	Prevention	Parents and caregivers	Awareness of injury prevention and safety behavior at home, user experience of the app, and motivations for injury prevention and challenges in making one’s home safer		Cursory overview of literature on the delivery of preventive information	Parents or legal guardians (n=40)	5 focus groups, web-based pretest and posttest survey, and app use data	Thematic analysis and statistical techniques, for example, chi-square test

^a^GUS: Growing up Safely.

### Study Quality: MMAT Results

Of the 5 studies included, 2 (40%) exclusively applied qualitative methods such as focus groups, 2 (40%) used quantitative methods such as surveys, and the last study (n=1, 20%) used a mixed methods approach, as documented in the MMAT overview (Multimedia Appendix 6). In total, 60% (3/5) of the studies used usability constructs, such as perceived usefulness or functionality assessment, and let the participants test the app while conducting focus groups or gaining data via web-based surveys [48-50].

Critically appraising the methodological quality of the evaluation studies was challenged by a lack of reported details on data collection, data analysis, and the interpretation of the study results. The mixed methods study by Roberts et al [50] provided the most methodological details. On the basis of this paper, we were able to answer 14 of the 15 MMAT questions on methodological quality. In contrast, the studies by Iskander et al [46] and Richmond et al [49] were the most difficult to assess. In this case, 2 out of 5 questions from the MMAT could not be answered. Therefore, a valid assessment of the overall study quality is hardly possible, which is why we will only address a few specific issues in this section.

Most studies (4/5, 80%) used appropriate measures and methods to address their research interests; however, Litovitz et al [48] did not provide enough information for an assessment. No study used a representative sample of their target population, although they all recruited the study participants from the group of potential app users. Regarding data analysis, Iskander et al [46] and Richmond et al [49] provided insufficient information to assess the validity of the data analysis process and the interpretation of the data. However, as far as can be assessed, Jones et al [47], Litovitz et al [48], and Roberts et al [50] largely evaluated and interpreted the research data adequately.

Overall, the quality appraisal by means of the MMAT resulted in an average overall score of approximately 50%, indicating a rather moderate methodological quality of the included studies. However, the lack of reported details contributed to this low score to a considerable degree. This points to the difficulties related to the adequate assessment of a paper’s methodological quality. The MMAT is based on the assumption that publications present all methodological information in an ideal-typical way; however, this is not always the case. Accordingly, the average overall score reported previously should be treated with caution as, in all cases, no definite assessment could be made on at least 1 item.

### Answering the RQs

#### Mobile Apps Dealing With the Prevention and Handling of Unintentional Injuries in Children via Mobile Apps (RQ 1)

Each paper presented the evaluation of a single app. In total, 60% (3/5) of the apps focused on preventing unintentional injuries (*Grow up Safely*, *Active & Safe Central*, and *Make Safe Happen*), whereas the remaining 40% (2/5) offered advice on handling unintentional injuries through appropriate first aid measures (*Dental Trauma* first aid mobile app and *webPOISONCONTROL* app; Table 1).

#### App Architecture Characteristics and Features

In total, 60% (3/5) of the apps offer features that allow for tailoring the information to the respective user. In both *Grow up Safely* and *Make Safe Happen*, users can select the age of their child and then automatically receive the injury prevention information that is important for this phase of their child’s life. The *webPOISONCONTROL* app works in a similar way by providing personalized recommendations on how to react in case of an intoxication based on the child’s age and other personal information. To assist users in implementing preventive measures, *Make Safe Happen* and *webPOISONCONTROL* include reminders via calendar notifications or emails to encourage consistent safety behavior. In addition, *Make Safe Happen* provides checklists to assess injury hazards in one’s home. A barcode scanner in the *webPOISONCONTROL* app can help identify toxic substance ingredients. *Active & Safe Central*, *Make Safe Happen*, and the *webPOISONCONTROL* app also provide additional information via external links. A download function has been integrated into *Active & Safe Central* allowing resources, evidence synthesis tables, and reports to be saved.

The paper on the *Dental Trauma* app by Iskander et al [46] only provided very rudimentary information on what prevention or handling information was offered and how it was provided by the app (Table 2).

**Table 2:** Overview of app features and visual elements.

	Features						Visual elements		
	Information provision	Tailored information	Tools for hazard identification	Reminders or follow-up	Data download	Photographs		Infographics	Data tables
Dental Trauma first aid mobile app	✓					✓			
Grow up Safely	✓	✓				✓			
webPOISONCONTROL app	✓	✓	✓	✓		✓			
Active & Safe Central	✓				✓	✓		✓	✓
Make Safe Happen	✓	✓	✓	✓		✓			

##### Visualization Elements

The use of visualizations was only explained to a very limited extent. Although Roberts et al [50], Iskander et al [46], Litovitz et al [48], and Jones et al [47] included screenshots of the apps in their papers, they did not provide any details on how the pictures in the app were chosen or what purpose they served. On the basis of the screenshots, *Dental Trauma* used photographs to differentiate between injuries, whereas *Grow up Safely* and *Make Safe Happen* used screenshots mainly to illustrate the app and its menu. The *webPOISONCONTROL* screenshots indicated minimal use of visualizations, with the only photo showing 2 pills of a particular drug. *Active & Safe Central* used more visualizations, mentioning infographics and data tables illustrating the individual injury risks associated with different sports and recreational activities (Table 2).

##### Target Audience

All apps were designed to be used by parents or caregivers (Table 1). Medical staff or physicians were only indicated as target users for *Active & Safe Central,* which also aims to inform the public, mentioning parents, coaches, athletes, and injury researchers. Roberts et al [50] and Iskander et al [46] collected demographic information such as age, level of education, and ethnicity of the app users. However, only Iskander et al [46] included this information in their analysis of individual preferences. They found that age did not have an effect on the frequency of internet use or on the preference for health information being delivered via posters or apps. The authors noted that this might be due to the small age range of the caregivers represented in the study. Differences were found in the interest in using an app to inform oneself about (dental) health information being higher for people with a lower level of education. In this case, a lower level of education was associated with a higher interest in information. The authors suggested that this might be due to a higher baseline health knowledge in people with higher education levels [46].

#### Theoretical and Methodological Foundations of the Evaluative Study Designs (RQ 2)

##### Theoretical Foundations and Evidence Research

Although there are a number of established approaches to researching the acceptability and use of an app (eg, the Technology Acceptance Model [51] and Unified Theory of Acceptance and Use of Technology model [52]), these were not used in any of the studies. Furthermore, no existing theories were applied in the development of the apps. However, 20% (1/5) of the papers mentioned *health literacy* as relevant to (visual) language [47] in the discussion part, whereas another research team used “an integrated knowledge translation approach” [49] but did not reference previous research on this approach or describe how they operationalized it. Therefore, 80% (4/5) of the studies did not follow a theory-based research approach, whereas the fifth paper did not provide sufficient information on how the chosen approach was translated into methodology.

Existing evidence-based knowledge on how best to communicate prevention or handling information to caregivers via mHealth apps (eg, simple language, visuals, and examples) was not systematically considered in any of the 5 included articles (Table 1). However, Richmond et al [49] and Jones et al [47] thematically reviewed the literature on unintentional injuries in children focusing on the type of injury and the percentage of accidents.

##### Assessed Variables and Methodological Foundations

Despite the lack of a common theoretical background, all studies (5/5, 100%) evaluated at least 1 variable related to usability or user experience, although this was evaluated in varying degrees of detail. Jones et al [47] and Richmond et al [49] also collected data on the evaluation of the apps’ look and appearance. User-related variables were also included in all studies (5/5, 100%). Table 3 provides an overview of the variables that were not only mentioned but also analyzed.

**Table 3:** Overview of variables.

Study	App-related variables						User-related variables		
	Usability or user experience	Look or appearance	Functionality	Interactivity or navigation	Issues or limitations	Awareness or knowledge		Attitudes or motivations	Demographics
Iskander et al [46]	✓					✓		✓	✓
Jones et al [47]	✓	✓				✓			
Litovitz et al [48]	✓		✓					✓	
Richmond et al [49]	✓	✓	✓	✓	✓			✓	
Roberts et al [50]	✓					✓		✓	

The variety of the assessed variables is also reflected in the different methods used in the studies, ranging from simple to rather complex designs. The most commonly used qualitative method was focus groups to evaluate the app either during or after development [47,49,50]. Jones et al [47] and Richmond et al [49] examined the interaction of users with the app, whereas Roberts et al [50] focused more on the participants’ attitudes. The most common quantitative method used in the analyzed studies was surveys. These were also used at different points in time within the study design—Iskander et al [46] only surveyed participants after they had used the app, whereas Roberts et al [50] used a pre- and posttest design to assess the extent to which the app influenced safety behavior. This study also included an analysis of the app usage data regarding frequency of use and retrieved information. In all studies (5/5, 100%), participants were recruited from the target group of app users, mainly parents or caregivers of young children.

#### Needs of Private Caregivers, Professional Caregivers, and Health Professionals and Constraints Preventing the Use of the App (RQ 3)

##### Overview

As mentioned previously, all studies (5/5, 100%) focused on private caregivers (eg, parents and legal guardians), although Litovitz et al [48] offered no detailed description of the study participants. No study explicitly involved professional caregivers or health professionals in the evaluation of the app except for Richmond et al [49], who included “injury researchers and practitioners” as well as end users [49], and Litovitz et al [48], who asked a toxicologist to analyze the safety of the triage recommendations. Jones et al [47] mentioned parents and “carers” but did not explain who was considered a carer and only included mothers in their sample.

Only 40% (2/5) of the papers offered results on the needs of the study participants. Jones et al [47] provided a detailed report on participants’ needs based on several focus group discussions, whereas Litovitz et al [48] only briefly summarized the most liked or preferred features that were collected through a web-based survey via open comment fields. Both papers mentioned trust as a relevant factor. However, without further elaboration, Litovitz et al [48] indicated the need for trusted and accurate information sources, whereas Jones et al [47] found trust to be an influencing factor for app use, with participants not only demanding links to other reputable sources in the app but also feeling “more likely to use the app if it had been recommended by a trusted source such as a health visitor or midwife” [47]. Data security and privacy were only reported in the study by Litovitz et al [48].

Considering the contents of the app, the participants expressed their need for simple, easy-to-understand texts that offered more than trivial or “common sense” information [47]. This need for simplicity was also emphasized in the study by Litovitz et al [48] but not discussed further. Instead, Litovitz et al [48] listed several other needs—such as helpfulness, convenience, or a step-by-step approach with regard to their app—without further elucidating them. Visuals were generally evaluated as relevant in the study by Jones et al [47]; however, the participants expressed a dislike of images of wounds or other injuries, stating that they might distract users from the text or context.

##### Constraints of App Use

Jones et al [47] and Roberts et al [50] also identified several aspects that might prevent either app use or the implementation of new prevention behaviors. The main constraint for app use is a lack of awareness of the app (eg, as the users forget to access the app regularly after downloading it). Push notifications that promote regular engagement with the app were seen as an adequate tool to combat this constraint [47]. However, even if users engage with the app and receive relevant information through it, this is no guarantee that they will also follow the app’s recommendations. This might be due to being overwhelmed by a large number of recommended actions, a lack of resources to implement new safety measures, or disagreements with other family members regarding their appropriateness [50].

## Discussion

### Principal Findings

This systematic review is the first to focus exclusively on mHealth interventions aimed at the prevention *and* handling of unintentional injuries in children aged <7 years as well as the methodology applied in these evaluation studies. The results show that a theory- and method-driven evaluation of app usability is rarely applied. This hampers the comparability of evaluations of different apps with regard to user preferences and target group-specific adaptation.

Previous reviews have assessed the effectiveness of preventive interventions regarding child injuries in general [11,13], examined the state of research in relation to low- and middle-income countries [53] or disadvantaged groups [54], or focused on technology-based interventions without specifically addressing private and professional caregivers [22]. Although unintentional injuries cause pain, disabilities, and death in children and, therefore, pose a major public health challenge [53,55,56], not many apps deal with the prevention or handling of unintentional child injuries. Accordingly, research on these apps is limited. We identified 5 papers that did analyze existing apps on this issue and evaluated them. However, the excluded conference abstracts [22,57] and study protocols [58,59] indicate that more research is being conducted. Cooray et al [57] proved this with their article published in 2021 on a behavioral theory-based app for parents to prevent falls in children. They emphasized that their methodological approach was the first of its kind, driven by the same rationale on which our review is based. That study shows that a theory-based, evidence-based, and user-centered approach to digital app development is a useful way to intentionally affect the behavior of the target group. However, as their article was published after our data collection period, it was not included in our review.

In addition, we identified studies on a theoretical framework for developing such an app; however, they did not include the development of the mobile app itself [25,60].

All 5 included studies explored factors of app usability; however, none of them drew on findings from evaluation research on mHealth technologies. In addition, the study designs focused on an exploratory or inductive approach instead of relying on existing knowledge that explains use or health information–seeking behavior. Even though data on individual variables were collected, these were considered independently of health behavior theories or models as a research framework.

This isolated examination of single variables reduces the validity of the studies. By drawing on proven theories and models, it would have been possible to systematically test central basic assumptions whose scope exceeds the specific study context. In contrast, without reference to existing theories and models, it is not clear whether the findings of a study represent an isolated phenomenon or whether they point to generalizable findings.

Moreover, even before the communicative content is developed, different theories can provide clues as to which variables, such as the users’ attitudes, subjective norms, or perceived self-efficacy, influence their behavior. This knowledge can help develop more effective communication messages for different target groups.

As previous usability studies in this specific research field have not been linked to mobile information behavior models or constructs such as health literacy, the applied research on mHealth interventions with regard to unintentional injuries in children is rather rudimentary (for similar findings on mobile apps in general, see the study by Chib and Lin [23]).

An explanatory factor could be that most researchers on this topic work in medical fields and, therefore, might not be familiar with information behavior models from a communication science perspective. An interdisciplinary look at the development and evaluation of such apps could advance research in this area.

Another point concerns the publishing strategies for scientific publications. In the study by Richmond et al [49], we can see that another publication by this research team [60] describes in much more detail how their framework for possible intervention strategies was developed “emphasizing four types of research evidence [...]: (1) epidemiologic evidence describing the burden and cause of injury, (2) evidence concerning the effectiveness of interventions, (3) evidence on effective methods for implementing promising interventions at a population level, and (4) evidence and theory from the behavioral sciences” [60]. This shows that knowledge from different disciplines did indeed play a role in the development of the mobile apps. However, their 2015 paper had to be excluded as it did not meet our inclusion criteria given that there was no mobile app developed or evaluated.

However, the included 2019 paper only mentions this information in passing without explaining how the different types of evidence influenced the implementation and transformation of scientific knowledge on sports injuries and prevention. Our review would have needed both papers to provide a complete picture of the current state of the art (see the *Strengths and Limitations* section). Such publication strategies need to be considered for future reviews.

Although the papers we analyzed did not have much in common, two categories could be identified among the examined variables: (1) app-related variables describing, for example, ease of use, and (2) user-related variables such as knowledge or risk perception. However, these papers did not provide information on how these variables were operationalized in the surveys [46,48,50]. This is a general challenge when researchers have to describe their study in a very limited format. Therefore, the methodological section should become a more important chapter in journal articles so that the methods used to obtain the data can be reflected upon. Only then will it be possible to evaluate the quality of a study and check the possibilities of reproducing it. This not only helps promote good scientific practice but can also strengthen trust in science.

### Current Gaps

Usability is not just about the presentation of knowledge but also deals with ergonomic design, user-friendly settings, and accessibility (eg, clear layout, icons and their comprehensibility, and understandable error messages [61]) as well as technical setting options. Although some of the studies (2/5, 40%) evaluated aspects such as the layout of the apps, icon selection, and comprehensibility, they did not use a comprehensive concept of software ergonomics [62].

Regarding visualizations, the studies showed somewhat conflicting results [49]. On the one hand, the data proved that images help users navigate and search within apps. In contrast, especially in the area of injury management, images of injuries can be stressful. This fine line between the positive and negative effects of using images in health apps needs to be handled with great care.

Health literacy was implicitly addressed in the work by Jones et al [47]. In this case, the study participants discussed whether the texts in the app were easy and quick to understand. Nevertheless, the content should still be interesting enough to hold the readers’ attention. For app developers, it could be a challenge to address a wider range of health literacy (see the *Constraints of App Use* section).

Linked to this are the needs of the target users—although some apps (eg, *Make Safe Happen* [50]) distinguished very precisely between different groups of parents (eg, based on the age of the child), others had rather vague target groups [49]. Targeted communication is essential if the mHealth intervention intends to make the information relevant to the specific target groups—considering data on their knowledge, (risk) perceptions, and motivations [63]. Furthermore, contrary to our expectations, only private caregivers were examined in more detail. Even though health professionals were not addressed as target users—except in the study by Richmond et al [49]—their views and reflections might offer relevant insights, especially as they are an effective intermediary [13] in the prevention and handling of unintentional child injuries.

Interestingly enough, gender did not play an analytical role, even though research shows that especially mothers of young children, expectant mothers [64,65], and mothers with low health literacy [66] use mHealth communication.

At least one study was able to show that users pay attention to (1) sources that recommend the app or (2) whether the app quotes, mentions, or links to reputable sources (eg, well-known people such as midwives [47]). This means that it is advisable for app creators not only to obtain the information in the app from evidence-based or familiar sources and relevant institutions but also to clearly mark the sources in the app.

### Constraints of App Use

We have already mentioned how difficult it is for app content developers to consider different health literacy levels and demands. This includes not only the step of accessing information but also of understanding, appraising, and applying it [67]. One way to give people access to information via mobile apps could be push notifications. In particular, injury prevention apps should be designed to remind the user to consult the app in situations of uncertainty. In this case, too, the needs of the target groups are decisive for the design of the app, be it the ergonomics or the content. The digital possibilities of mHealth interventions to support behavior change or informed decision-making are present [68], but so are barriers. The amount of information may be sufficient for one user but too much for another—even in the same target group. These aspects cannot be easily generalized for every app. It depends on the topic, the individual user, whether the amount of information is appropriate, and many other aspects. In short, theory-based evaluations are crucial. This also applies to the question of whether and how app use leads to changes in behavior or has an impact on prevention or first aid behavior [23]. None of the studies evaluated the effects of app use. This highlights the importance of continuous app evaluation to match the intended use with the actual app use.

### Strengths and Limitations

One of the assumed strengths of the review later turned out to be a weakness as well: the scope of this review was quite narrow, which led to a very limited number of results.

However, this particular limitation may also indicate one of its strengths. The function of a literature review may lie not only in its identification, analysis, or synthesis of relevant research literature but also in its ability to identify desiderata or research gaps in a specific research area. Therefore, the study not only contributes to the investigation of existing studies’ deficits regarding their generalizability but also links the results to extant theories or models. The literature review might also help uncover previously unconsidered aspects or variables that could benefit future studies in the field of mobile apps dealing with the prevention and handling of unintentional injuries in children. At the same time, a desiderata-oriented form of literature review might also help in identifying common deficits in publication and writing processes that so far have often been overlooked.

Concerning the search method of our systematic review, a further limitation should be noted. In its third pillar, the developed search string only comprises generalized categories of unintentional injuries, which were identified by means of a prereview mapping as well as by recourse to the definition of unintentional injuries offered by Sleet [9] and Schnitzer [10]. Thus, this operationalization of unintentional injuries only covers categories of unintentional injuries (such as burns or poisonings) but not the concrete or possible causes of such accidents (such as stoves and ovens or a specific chemical) as, otherwise, the search string would have had to define an all-encompassing, expansive list of possible causes covering every conceivable scenario (eg, poisoning through medications, alcohol, and hydrocarbons). Therefore, the developed search string cannot fully ensure that all the relevant papers were actually identified. In at least 1 known case [69], a relevant article was not identified because it did not refer to specific categories of unintentional injuries in the title, abstract, or keywords.

Owing to the number of articles, the titles and abstracts of 129 articles were checked by only 1 rater each. To keep potential bias to a minimum, the interrater reliability was examined beforehand by calculating the Fleiss κ. In case of uncertainty, the inclusion or exclusion of an article was discussed among the researchers.

As we excluded all papers that did not explicitly report an empirical study on the evaluation of an app, we were not able to include papers that solely discussed the theoretical frameworks of app development. However, when designing the review, we did not consider that researchers might publish the results of their work in several articles (eg, 1 focusing on theoretical [60] and 1 on empirical [49] aspects). This turned out to limit the validity of our findings, especially our conclusions, as we were not able to include all published information on a particular research project.

### Conclusions and Directions for Future Research

The aim of this systematic review was to summarize the state of the art on the evaluation of mHealth apps in the context of the handling and prevention of unintentional injuries in children aged <7 years. The findings of this review highlight two objectives that play an important role in developing and evaluating these apps: (1) the use of tailored (visualized) information being a part of (2) knowledge transfer and transformation. Both of these objectives play a role when it comes to app- and user-related variables.

Information tailoring in mHealth contexts contains more than the message itself; the app allows for personalization features such as the age of the child that can make the provided content more useful for the user [47]. Future research should combine insights into user experience and usability with existing theories on mobile information behavior and (visual) literacy constructs [14]. Thus, use and health information-seeking behavior variables may deliver data on how to tailor information to the needs of different target groups, including professionals. As previous research shows, gender should also be considered as a relevant predictor of mHealth app use [70]. When all this is considered, the efficiency of an mHealth intervention can be increased.

On the basis of this review, it is concluded that the focus of evaluative usability studies on the prevention and handling of unintentional injuries in children should be shifted from technical developments and first-phase studies examining singular variables to collecting evaluation data derived from theories and models to raise the validity of the foundational premises measuring health behavior.

## Appendix

Multimedia Appendix 1 PRISMA (Preferred Reporting Items for Systematic Reviews and Meta-Analyses) 2020 checklist.

Multimedia Appendix 2 Research questions and literature search process.

Multimedia Appendix 3 Inclusion and exclusion criteria.

Multimedia Appendix 4 PRISMA (Preferred Reporting Items for Systematic Reviews and Meta-Analyses) 2020 flowchart.

Multimedia Appendix 5 Code system.

Multimedia Appendix 6 Results of the Mixed Methods Appraisal Tool.

## Abbreviations

mHealth: mobile health
MMAT: Mixed Methods Appraisal Tool
PRISMA: Preferred Reporting Items for Systematic Reviews and Meta-Analyses
RQ: research question

## References

[1] Saß A-C, Kuhnert R, Gutsche J (2019). [Unintentional injuries in childhood and adolescence-prevalence, locations, and mechanisms: results from KiGGS Wave 2 and trends]. Bundesgesundheitsblatt - Gesundheitsforschung - Gesundheitsschutz.

[2] Le Roux G, Sinno-Tellier S, Descatha A, French Poison Control Centre members (2020). COVID-19: home poisoning throughout the containment period. Lancet Public Health.

[3] Bressan S, Gallo E, Tirelli F, Gregori D, Da Dalt L (2021). Lockdown: more domestic accidents than COVID-19 in children. Arch Dis Child.

[4] Ferrer IL, Baptistella MK, Oliveira FN, Souza AG, Cunha LC, Magalhães AF (2021). Poisoning in children and adolescents assisted during the COVID-19 pandemic at the Toxicological Information and Assistance Center in Federal District, Brazil (CIATOX -DF): descriptive, cross-sectional, and analytical study with 1.037 patients. Res Soc Dev.

[5] Gielen AC, Bachman G, Badaki-Makun O, Johnson RM, McDonald E, Omaki E, Pollack Porter KM, Ryan L, Shields W (2020). National survey of home injuries during the time of COVID-19: who is at risk?. Inj Epidemiol.

[6] Mahdavi SA, Kolahi A-A, Akhgari M, Gheshlaghi F, Gholami N, Moshiri M, Mohtasham N, Ebrahimi S, Ziaeefar P, McDonald R, Tas B, Kazemifar AM, Amirabadizadeh A, Ghadirzadeh M, Jamshidi F, Dadpour B, Mirtorabi SD, Farnaghi F, Zamani N, Hassanian-Moghaddam H (2021). COVID-19 pandemic and methanol poisoning outbreak in Iranian children and adolescents: a data linkage study. Alcohol Clin Exp Res.

[7] Palmer CS, Teague WJ (2021). Childhood injury and injury prevention during COVID-19 lockdown - stay home, stay safe?. Injury.

[8] Sahar AH, Hasnae H, Hajar M, Mohammed C, Sana C, Sanae A, Ilham T, Moustapha H (2021). Children’s poisoning profile during the Covid-19 pandemic – experience of Hassan II University Hospital in Fez, MOROCCO. Proceedings of the International Congress on Health Vigilance (VIGISAN 2021).

[9] Sleet DA (2018). The global challenge of child injury prevention. Int J Environ Res Public Health.

[10] Schnitzer PG (2006). Prevention of unintentional childhood injuries. Am Fam Physician.

[11] Jullien S (2021). Prevention of unintentional injuries in children under five years. BMC Pediatr.

[12] Zhao J, Freeman B, Li M (2016). Can mobile phone apps influence people's health behavior change? An evidence review. J Med Internet Res.

[13] Stehr P, Reifegerste D, Rossmann C, Caspar K, Schulze A, Lindemann A-K (2022). Effective communication with caregivers to prevent unintentional injuries in children under seven years. A systematic review. Patient Educ Couns.

[14] Kreps GL (2017). The relevance of health literacy to mHealth. Stud Health Technol Inform.

[15] Nacinovich M (2013). Defining mHealth. J Commun Healthc.

[16] Kreps GL (2017). Online information and communication systems to enhance health outcomes through communication convergence. Hum Commun Res.

[17] Larson RS (2018). A path to better-quality mHealth apps. JMIR Mhealth Uhealth.

[18] Sackett DL, Rosenberg WM, Gray JA, Haynes RB, Richardson WS (1996). Evidence based medicine: what it is and what it isn't. BMJ.

[19] Hirschberg I, Seidel G, Strech D, Bastian H, Dierks M-L (2013). Evidence-based health information from the users’ perspective – a qualitative analysis. BMC Health Serv Res.

[20] Stehr P, Heinemeier D, Rossmann C (2018). Evidenzbasierte | evidenzinformierte Gesundheitskommunikation.

[21] Lupton D (2016). Towards critical digital health studies: reflections on two decades of research in health and the way forward. Health (London).

[22] Omaki E, Rizzutti N, Shields W, Zhu J, McDonald E, Stevens MW, Gielen A (2017). A systematic review of technology-based interventions for unintentional injury prevention education and behaviour change. Inj Prev.

[23] Chib A, Lin SH (2018). Theoretical advancements in mHealth: a systematic review of mobile apps. J Health Commun.

[24] Cho Y-M, Lee S, Islam SM, Kim S-Y (2018). Theories applied to m-health interventions for behavior change in low- and middle-income countries: a systematic review. Telemed J E Health.

[25] Ning P, Gao D, Cheng P, Schwebel DC, Wei X, Tan L, Xiao W, He J, Fu Y, Chen B, Yang Y, Deng J, Wu Y, Yu R, Li S, Hu G (2019). Needs analysis for a parenting app to prevent unintentional injury in newborn babies and toddlers: focus group and survey study among Chinese caregivers. JMIR Mhealth Uhealth.

[26] Schnall R, Higgins T, Brown W, Carballo-Dieguez A, Bakken S (2015). Trust, perceived risk, perceived ease of use and perceived usefulness as factors related to mHealth technology use. Stud Health Technol Inform.

[27] Sowon K, Chigona W (2020). Trust in mHealth: how do maternal health clients accept and use mHealth interventions?. Proceedings of the Conference of the South African Institute of Computer Scientists and Information Technologists 2020.

[28] Nolen SL, Giblin-Scanlon LJ, Boyd LD, Rainchuso L (2018). Development and testing of a smartphone application prototype for oral health promotion. J Dent Hyg.

[29] Carroll AL, Christian R, Palokas M (2022). Mobile injury prevention programs for children: a scoping review protocol. JBI Evid Synth.

[30] Xiao Y, Watson M (2017). Guidance on conducting a systematic literature review. J Plan Educ Res.

[31] Page M, McKenzie JE, Bossuyt PM, Boutron I, Hoffmann TC, Mulrow CD, Shamseer L, Tetzlaff JM, Akl EA, Brennan SE, Chou R, Glanville J, Grimshaw JM, Hróbjartsson A, Lalu MM, Li T, Loder EW, Mayo-Wilson E, McDonald S, McGuinness LA, Stewart LA, Thomas J, Tricco AC, Welch VA, Whiting P, Moher D (2021). The PRISMA 2020 statement: an updated guideline for reporting systematic reviews. Syst Rev.

[32] Lee S, Koubek RJ (2010). Understanding user preferences based on usability and aesthetics before and after actual use. Interact Comput.

[33] Armstrong R, Jackson N, Doyle J, Waters E, Howes F (2005). It's in your hands: the value of handsearching in conducting systematic reviews of public health interventions. J Public Health (Oxf).

[34] Richards D (2008). Handsearching still a valuable element of the systematic review. Evid Based Dent.

[35] (2008). iPhone App Store downloads top 10 million in first weekend. Apple.

[36] Ellsäßer G (2017). Unfälle, Gewalt, selbst verletzung bei Kindern und Jugendlichen Ergebnisse der amtlichen Statistik zum Verletzungsgeschehen 2014. Statistisches Bundesamt.

[37] Brussoni M, Brunelle S, Pike I, Sandseter EB, Herrington S, Turner H, Belair S, Logan L, Fuselli P, Ball DJ (2015). Can child injury prevention include healthy risk promotion?. Inj Prev.

[38] Ellsäßer G, Trost-Brinkhues G, Albrecht M (2014). [Injury prevention in young children]. Bundesgesundheitsblatt – Gesundheitsforschung – Gesundheitsschutz.

[39] Gisev N, Bell JS, Chen TF (2013). Interrater agreement and interrater reliability: key concepts, approaches, and applications. Res Social Adm Pharm.

[40] McHugh ML (2012). Interrater reliability: the kappa statistic. Biochem Med.

[41] Belur J, Tompson L, Thornton A, Simon M (2018). Interrater reliability in systematic review methodology: exploring variation in coder decision-making. Sociol Methods Res.

[42] Landis JR, Koch GG (1977). The measurement of observer agreement for categorical data. Biometrics.

[43] Wohlin C (2014). Guidelines for snowballing in systematic literature studies and a replication in software engineering. Proceedings of the 18th International Conference on Evaluation and Assessment in Software Engineering.

[44] Hong QN, Pluye P, Fàbregues S, Bartlett G, Boardman F, Cargo M, Dagenais P, Gagnon M-P, Griffiths F, Nicolau B, O'Cathain A, Rousseau M-C, Vedel I (2019). Improving the content validity of the mixed methods appraisal tool: a modified e-Delphi study. J Clin Epidemiol.

[45] Campbell JL, Quincy C, Osserman J, Pedersen OK (2013). Coding in-depth semistructured interviews: problems of unitization and intercoder reliability and agreement. Sociol Methods Res.

[46] Iskander M, Lou J, Wells M, Scarbecz M (2016). A poster and a mobile healthcare application as information tools for dental trauma management. Dent Traumatol.

[47] Jones F, Whitehouse A, Dopson A, Palaghias N, Aldiss S, Gibson F, Shawe J (2020). Reducing unintentional injuries in under fives: development and testing of a mobile phone app. Child Care Health Dev.

[48] Litovitz T, Benson BE, Smolinske S (2016). webPOISONCONTROL: can poison control be automated?. Am J Emerg Med.

[49] Richmond SA, Black AM, Jacob J, Babul S, Pike I (2019). 'Active & Safe Central': development of an online resource for the prevention of injury in sport and recreational activity. Inj Prev.

[50] Roberts KJ, McAdams RJ, Kristel OV, Szymanski AM, McKenzie LB (2019). Qualitative and quantitative evaluation of the make safe happen app: mobile technology-based safety behavior change intervention for parents. JMIR Pediatr Parent.

[51] Davis FD (1989). Perceived usefulness, perceived ease of use, and user acceptance of information technology. MIS Q.

[52] Venkatesh V, Morris MG, Davis GB, Davis FD (2003). User acceptance of information technology: toward a unified view. MIS Q.

[53] Tupetz A, Friedman K, Zhao D, Liao H, Isenburg MV, Keating EM, Vissoci JR, Staton CA (2020). Prevention of childhood unintentional injuries in low- and middle-income countries: a systematic review. PLoS One.

[54] Möller H, Falster K, Ivers R, Jorm L (2015). Inequalities in unintentional injuries between indigenous and non-indigenous children: a systematic review. Inj Prev.

[55] (2018). Burns. World Health Organization.

[56] (2022). Drowning. World Health Organization.

[57] Cooray N, Sun S, Adams S, Elkington J, Ho C, Keay L, Nassar N, Brown J (2021). Developing behavioural theory grounded and user-centred mobile app to prevent infant falls. Injury Prevent.

[58] McKenzie LB, Roberts KJ, Clark R, McAdams R, Abdel-Rasoul M, Klein EG, Keim SA, Kristel O, Szymanski A, Cotton CG, Shields WC (2018). A randomized controlled trial to evaluate the Make Safe Happen® app-a mobile technology-based safety behavior change intervention for increasing parents' safety knowledge and actions. Inj Epidemiol.

[59] Omaki E, Shields WC, McDonald E, Aitken ME, Bishai D, Case J, Gielen A (2017). Evaluating a smartphone application to improve child passenger safety and fire safety knowledge and behaviour. Inj Prev.

[60] Chambers A, Richmond SA, Logan L, Macarthur C, Mustard CA (2015). The development of a framework to integrate evidence into a national injury prevention strategy. J Public Health (Oxf).

[61] Yong TS, Perialathan K, Ahmad M, Juatan N, Abdul Majid L, Johari MZ (2021). Perceptions and acceptability of a smartphone app intervention (ChildSafe) in Malaysia: qualitative exploratory study. JMIR Pediatr Parent.

[62] Bevan N, Carter J, Harker S, Kurosu M (2015). Iso 9241-11 revised: what have we learnt about usability since 1998?. Human-Computer Interaction: Design and Evaluation.

[63] Elling JM, De Vries H (2021). Influence of animation- versus text-based delivery of a web-based computer-tailored smoking cessation intervention on user perceptions. Eur J Health Commun.

[64] Hiebert B, Hall J, Donelle L, Facca D, Jackson K, Stoyanovich E (2021). "Let me know when I'm needed": exploring the gendered nature of digital technology use for health information seeking during the transition to parenting. Digit Health.

[65] Lupton D, Pedersen S (2016). An Australian survey of women's use of pregnancy and parenting apps. Women Birth.

[66] Manganello JA, Falisi AL, Roberts KJ, Smith KC, McKenzie LB (2016). Pediatric injury information seeking for mothers with young children: the role of health literacy and ehealth literacy. J Commun Healthc.

[67] Kaphingst KA, Kreuter MW, Casey C, Leme L, Thompson T, Cheng M-R, Jacobsen H, Sterling R, Oguntimein J, Filler C, Culbert A, Rooney M, Lapka C (2012). Health Literacy INDEX: development, reliability, and validity of a new tool for evaluating the health literacy demands of health information materials. J Health Commun.

[68] Sartori F, Savi M, Talpini J (2022). Tailoring mHealth apps on users to support behavior change interventions: conceptual and computational considerations. Appl Sci.

[69] Gielen AC, Bishai DM, Omaki E, Shields WC, McDonald EM, Rizzutti NC, Case J, Stevens MW, Aitken ME (2018). Results of an RCT in two pediatric emergency departments to evaluate the efficacy of an m-health educational app on car seat use. Am J Prev Med.

[70] Bol N, Helberger N, Weert JC (2018). Differences in mobile health app use: a source of new digital inequalities?. Inform Soc.

