# Supplementary figures and images for: Mobile Apps Aimed at Preventing and Handling Unintentional Injuries in Children Aged <7 Years: Systematic Review
Source: Interact J Med Res. 2023 Sep 6;12:e45258. doi: 10.2196/45258 (PMC10512123; doi:10.2196/45258)

## Multimedia Appendix 2

### Research Questions and Literature Search Process

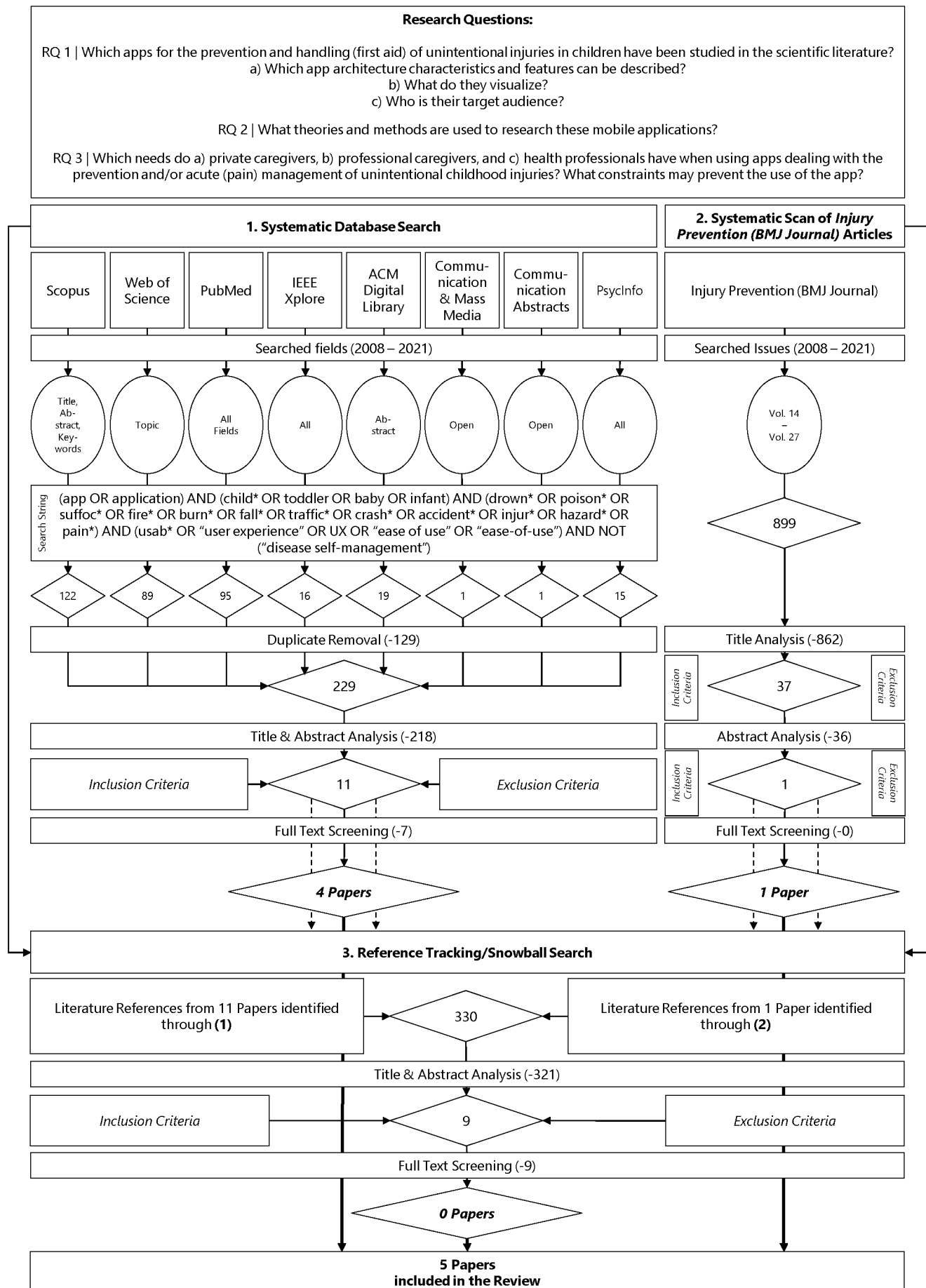

Supplement: Multimedia Appendix 2 [file ijmr_v12i1e45258_app2.pdf]

Multimedia Appendix 4  
PRISMA 2020 Flowchart

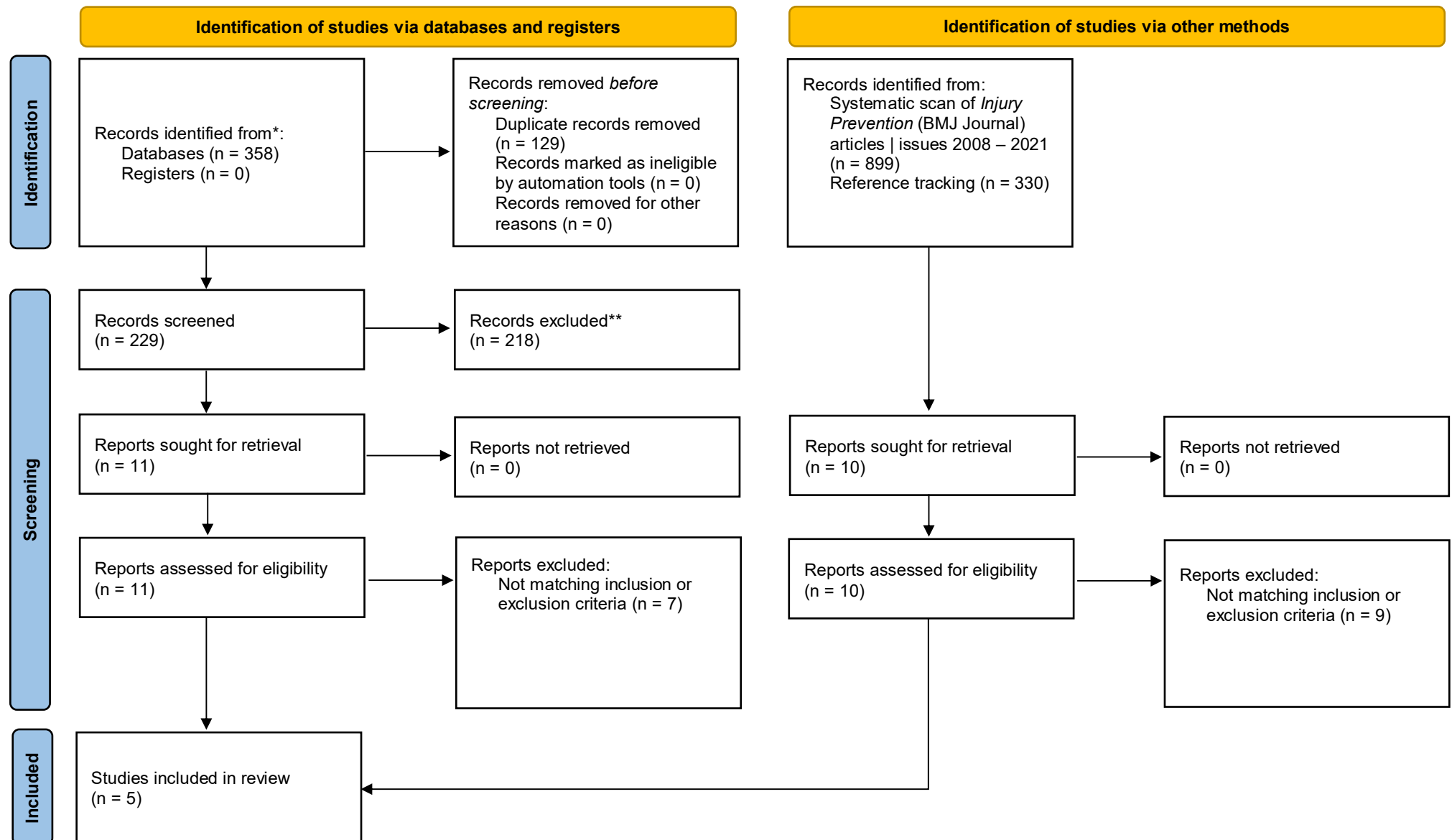

Supplement: Multimedia Appendix 4 [file ijmr_v12i1e45258_app4.pdf]
