# Supplementary material for: Mobile Apps Aimed at Preventing and Handling Unintentional Injuries in Children Aged <7 Years: Systematic Review
Source: Interact J Med Res. 2023 Sep 6;12:e45258. doi: 10.2196/45258 (PMC10512123; doi:10.2196/45258)
Supplement: Multimedia Appendix 3 [file ijmr_v12i1e45258_app3.pdf]

Multimedia Appendix 3

Inclusion and Exclusion Criteria

	Criteria	Rationale
Inclusion criteria	**I1** Articles written in English or German	- Languages spoken by the research team
	**I2** Original research articles or brief reports in peer-reviewed journals or conference proceedings/papers	- Focus on original, substantiated research<br>- Conference proceedings especially relevant in computer science, design and engineering
	**I3** Publications published between 2008-2021	- Introduction of mobile applications in 2008 (launch of the Apple App Store)
	**I4** Publications have to elaborate on the elements of at least one of the main research questions regarding mobile applications dealing with prevention and/or handling of unintentional childhood injuries	- Overview on existing mobile applications and their characteristics and features<br>- Theoretical and methodological background to research them<br>- Needs and constraints of private or professional caregivers and health professionals
Exclusion criteria	**E1** The publication does not meet all of the inclusion criteria I1-I4	- Not in English or German<br>- Not an original research article or brief report; not peer-reviewed<br>- Published before 2008 or after 2021<br>- Does not provide answers to the research questions
	**E2** The mobile application deals with handling / prevention of childhood injuries that were caused intentionally	- Different nature of injuries (e.g. abuse, self-inflicted injuries)<br>- Other prevention measures and strategies needed
	**E3** The paper focuses on education of medical staff / students	- Training situations not comparable with real live experiences
	**E4** The paper focuses on tracking/collecting individual health data (e.g. monitoring patients, self-management of diseases) as part of an ongoing treatment	- Different objectives compared to first aid and prevention<br>- Different target population for the app (esp. self-management)
	**E5** If the article only mentions a mobile application to illustrate something	- Paper needs to address the main research questions (see I4)
	**E6** If the paper provides no empirical data, e.g. by focusing only on technical / theoretical aspects	- Paper needs to contain original empirical research
	**E7** Articles to which we had no full access through databases or by contacting the corresponding author	
