# Supplementary material for: Mobile Apps Aimed at Preventing and Handling Unintentional Injuries in Children Aged <7 Years: Systematic Review
Source: Interact J Med Res. 2023 Sep 6;12:e45258. doi: 10.2196/45258 (PMC10512123; doi:10.2196/45258)
Supplement: Multimedia Appendix 5 [file ijmr_v12i1e45258_app5.pdf]

Multimedia Appendix 5

Code System

List of Codes	Frequency
Code system	724
● Bibliographic data	99
● Type of publication	6
● Author	24
● Title	5
● Year of publication	6
● Journal / conference (proceedings)	6
● Affiliation	20
● Other	1
● Tech	1
● Health	0
● General medicine / other medicine	13
● Pediatrics	5
● Country	12
● Challenges	38
● Public health challenges	0
● Evaluation	1
● Data / information acquisition	12

Multimedia Appendix 5

Code System

● Resources	4
● Injury circumstances	8
● Challenges of treatment	0
● Access to medical specialists	1
● Pain	0
● Injury itself	6
● Technological challenges	6
● Demand	1
● Accessibility	4
● Financial issues	1
● App description	0
● App elements	0
● Visual elements	8
● Tailored information	20
● Identify injury hazards	0
● Scanner / barcode entry	2
● Checklist	3
● Providing information / content	0
●External links	3

Code System

● Recommendations	15
● Data download	2
● Reminders / follow-ups	3
● App topic	0
● Objectives	24
● Technological objectives	9
● Accessibility	4
● Inclusiveness	1
● Efficiency / effectiveness	3
● Knowledge increase	2
● Tailor-made information	1
● Reliability / improve accuracy of judgement	2
● Public health objectives	15
● Evidence-informed policies	2
● Improve accessibility	4
● Increasing knowledge & awareness	8
● Injury reduction	1
● Affected children	7
● Target users	0

Code System

● The public	2
● Parents & caregivers	7
● Medical staff	1
● Patients	0
● Type of injury / pain	0
●Injury	0
● Specific	5
● General	2
● Pain	0
● Development of the app	0
● Developers	23
● Public organisations	3
● Medical institutions	7
● Universities	4
● Companies	2
● Users and / or public	7
● NGOs	6
● Development process	0
● Pilot project	1

Multimedia Appendix 5

Code System

● Development prior app manifestation	7
● Other	2
● Stakeholder involvement	0
● Others	4
● Target users	4
● Study design	0
● Theoretical base (models, effects, constructs)	1
● Research questions / objectives	11
● Methods	0
● Data collection methods	23
● Quantitative	0
● Survey	12
● App utilisation	5
● Qualitative	0
● Focus groups	8
● Interview / open ended questions	2
● Participatory observation	2
● Data analysis methods	22
●Quantitative	6

Code System

● Qualitative	4
● Triangulation	1
● Study population	0
● Sample of the study population / sample description	6
● Geographical location	7
● Demographics	0
● Role	0
● Developers or development-associated actors	1
● Other	7
● "Origin"	4
● Gender	5
● Age	6
● Sample size	6
● Evaluation / empirical testing	0
● Variables	0
● App	0
● Issues / limitations (eg, concerning data)	2
● Functionality	2
● Usability / user experience	12

Multimedia Appendix 5

Code System

● Interactivity / navigation	3
● Look / appearance / aesthetics	6
● User	12
● Attitudes / motivations	7
● Awareness / knowledge / previous experience	4
● Demographics	1
● Results / outcome	0
● Feedback on tailoring information	2
● Feedback on usability	1
● Feedback on safety of app recommendations	1
● Study participants	94
● Not identifiable	22
● Health professionals	7
● Professional caregiver	0
● Private caregiver	65
● Barriers or facilitators to safety behaviour or app use	0
● Barriers to safety behaviour: feeling overwhelmed	1
● Barriers to app use: unfamiliarity of features	2
● Facilitator to safety behaviour: awareness / knowledge	2

Multimedia Appendix 5

Code System

● Facilitator to safety behaviour: risk perception	1
● Barriers to safety behaviour: social environment	1
● Facilitator to app use: trust	3
● Barriers to app use: being unaware of the app	2
● Barriers to safety behaviour / app use: resources	5
● User needs / criticism / ideas for improvement	5
● Visual support but avoiding distressing images	1
● Not all features used (negative preferences)	2
● Criticism: avoiding trivial information	1
● Simple language	3
● Needs: trust / credibility	1
● Needs: clear communication of the purpose	1
● Needs: additional resources	3
● Influences of demographic variables	11
● General evaluation	9
● Behavioural (intention) change	7
● Learning and awareness	9
● Emotions associated with app usage	5
● Feedback on appearance / visualisations	2

Code System

● Feedback on style of language / understandability	3
● Feedback on presentation / layout	1
● App efficiency / speed	4
● Limitations	20
● Application	2
● Study	0
● Study design	4
● Sample	5
● Biases	3
● Missing / incomplete data	4
● Outlook	0
● Future research	0
● Warning	1
● Exploration	1
● Evaluation	3
● Future use	0
● Relevance	9
● App maintenance	1
● App improvement	0

Multimedia Appendix 5

Code System

● Add resources	2
● Add features or content	7
