# Supplementary material for: Mobile Apps Aimed at Preventing and Handling Unintentional Injuries in Children Aged <7 Years: Systematic Review
Source: Interact J Med Res. 2023 Sep 6;12:e45258. doi: 10.2196/45258 (PMC10512123; doi:10.2196/45258)
Supplement: Multimedia Appendix 6 [file ijmr_v12i1e45258_app6.pdf]

Multimedia Appendix 6

Results of the Mixed Methods Appraisal Tool (MMAT)

Types of study design	Methodological quality criteria	Iskander et al. 2016	Jones et al. 2020	Litovitz et al. 2016	Richmond et al. 2019	Roberts et al. 2016
**Screening questions (for all types)**	S1. Are there clear research questions resp. objectives?	✓	✓	✓	✓	✓
	S2. Do the collected data allow to address the research questions/objectives?	✓	✓	✓	?	✓
**1. Qualitative**	1.1. Is the qualitative approach appropriate to answer the research question?	–	✓	–	✓	✓
	1.2. Are the qualitative data collection methods adequate to address the research question?	–	✓	–	✓	✓
	1.3. Are the findings adequately derived from the data?	–	?	–	?	✓
	1.4. Is the interpretation of results sufficiently substantiated by data?	–	✓	–	×	✓
	1.5. Is there coherence between qualitative data sources, collection, analysis and interpretation?	–	✓	–	?	✓
**2. Randomized controlled trials**	Not applicable	–	–	–	–	–
**3. Non-randomized studies**	3.1. Are the participants representative of the target population?	×	–	–	–	–
	3.2. Are measurements appropriate regarding both the outcome and intervention (or exposure)?	✓	–	–	–	–
	3.3. Are there complete outcome data?	✓	–	–	–	–
	3.4. Are the confounders accounted for in the design and analysis?	?	–	–	–	–
	3.5. During the study period, is the intervention administered (or exposure occurred) as intended?	?	–	–	–	–
**4. Quantitative descriptive**	4.1. Is the sampling strategy relevant to address the research question?	–	–	✓	–	✓
	4.2. Is the sample representative of the target population?	–	–	×	–	×
	4.3. Are the measurements appropriate?	–	–	?	–	✓
	4.4. Is the risk of nonresponse bias low?	–	–	×	–	?
	4.5. Is the statistical analysis appropriate to answer the research question?	–	–	✓	–	✓
**5. Mixed methods**	5.1. Is there an adequate rationale for using a mixed methods design to address the research question?	–	–	–	–	✓
	5.2. Are the different components of the study effectively integrated to answer the research question?	–	–	–	–	✓
	5.3. Are the outputs of the integration of qualitative and quantitative components adequately interpreted?	–	–	–	–	✓
	5.4. Are divergences and inconsistencies between quantitative and qualitative results adequately addressed?	–	–	–	–	×
	5.5. Do the different components of the study adhere to the quality criteria of each tradition of the methods involved?	–	–	–	–	✓
**Score**		40 %	80 %	40 %	40 %	80 %

Note: “✓“ *= yes; „×“ = no; „?“ = cannot tell; “–“ = not applicable*
